# Administration of cytokine-induced myeloid-derived suppressor cells ameliorates renal fibrosis in diabetic mice

**DOI:** 10.1186/s13287-018-0915-0

**Published:** 2018-07-04

**Authors:** Ching-Chuan Hsieh, Chun-Liang Lin, Jie-Teng He, Meihua Chiang, Yuhsiu Wang, Yu-Chin Tsai, Chien-Hui Hung, Pey-Jium Chang

**Affiliations:** 1 Graduate Institute of Clinical Medical Sciences, College of Medicine, Chang-Gung University, Taoyuan, Taiwan; 2 Department of Surgery, Chang-Gung Memorial Hospital, 6, Sec. West Chia-Pu Road, Pu-Zi City, Chiayi County 613 Taiwan; 3 Department of Nephrology, Chang-Gung Memorial Hospital, Chiayi, Taiwan; 4 Kidney and Diabetic Complications Research Team (KDCRT), Chang-Gung Memorial Hospital, Chiayi, Taiwan

**Keywords:** Myeloid-derived suppressor cells, Immunotherapy, Fibronectin, Diabetic nephropathy

## Abstract

**Background:**

Diabetes is a proinflammatory state. Fibrosis of the renal glomerulus is the most common cause of end-stage renal disease. Glomerulosclerosis is caused by the accumulation of extracellular matrix (ECM) proteins in the mesangial interstitial space. Mesangial cells are unique stromal cells in the renal glomerulus that form the vascular pole of the renal corpuscle along with the mesangial matrix. Myeloid-derived suppressor cells (MDSCs) are heterogeneous immature myeloid cells that rapidly expand to regulate host immunity during inflammation, infection, and cancer. High concentrations of granulocyte–macrophage colony-stimulating factor (GM-CSF) alone or in combination with other molecules represent the most common ex-vivo protocol for differentiating MDSCs from bone marrow or from peripheral blood mononuclear cells. In this study, we analyzed and characterized the functions of MDSCs under the influence of mouse mesangial cells (MMCs) in a hyperglycemic environment and investigated whether cytokine-induced MDSCs ameliorated renal glomerulosclerosis in diabetic mice.

**Methods:**

Cytokine-induced MDSCs were propagated from bone marrow cells cultured with mouse recombinant GM-CSF, IL-6, and IL-1β. Diabetic mice were induced with streptozotocin (STZ) and maintained at a blood glucose concentration exceeding 350 mg/dl. The ECM of the renal cortex and fibronectin expression of MMCs were analyzed through immunohistochemistry and western blotting. Arginase 1 and inducible NO synthase expressions of MDSCs were evaluated using quantitative reverse-transcriptase PCR. Cytokines released from MMCs were examined using a cytokine array assay.

**Results:**

MDSCs in the diabetic mice were redistributed from the bone marrow into peripheral organs. An increase in fibronectin production was also observed in the renal glomerulus. MMCs in vitro produced more fibronectin and proinflammatory cytokines, such as macrophage inflammatory protein-2, RANTES, and stromal-cell-derived factor-1, under hyperglycemic conditions. The adoptive transfer of cytokine-induced MDSCs into STZ-induced mice normalized the glomerular filtration rate to reduce the kidney to body weight ratio and decrease fibronectin production in the renal glomerulus, ameliorating renal fibrosis. These results demonstrate the anti-inflammatory properties of cytokine-induced MDSCs and offer an alternative immunotherapy protocol for the management of diabetic nephropathy.

**Conclusions:**

The application of cytokine-induced MDSCs provides a promising treatment for renal fibrosis and the prevention of diabetic nephropathy.

**Electronic supplementary material:**

The online version of this article (10.1186/s13287-018-0915-0) contains supplementary material, which is available to authorized users.

## Background

Type 1 diabetes (T1D) is an insulin-dependent disorder characterized by kidney failure, blindness, heart disease, and chronic ulcers [1]. Nephropathy is one of the adverse effects of diabetes and is the most common cause of end-stage renal disease worldwide [2]. Progression of diabetic nephropathy to end-stage kidney disease is mediated by a host of processes, but none is as important as the gradual, inevitable scarring of the renal glomerulus, known as glomerulosclerosis. Glomerulosclerosis in diabetic nephropathy is caused by accumulation of extracellular matrix (ECM) proteins in the mesangial interstitial space, resulting in fibrosis manifested by either diffuse or nodular changes [3]. Extraglomerular cells, such as bone marrow (BM)-derived mesangial cell progenitors [4] and macrophages [5], may contribute considerably to glomerulosclerosis in diabetic nephropathy.

Myeloid-derived suppressor cells (MDSCs) were described more than 20 years ago in patients with cancer [6–8], but their functional importance in the immune system has only recently been appreciated. In pathological conditions, such as cancer, infectious diseases, sepsis, trauma, BM transplantation, and some autoimmune disorders, a partial block in the differentiation of immature myeloid cells results in an expansion of MDSCs [9]. In mice, these cells are defined by surface expression of CD11b (Mac-1) and Gr-1 (Ly6G); however, MDSCs have still not been well characterized in humans owing to the lack of specific markers [10]. Immunoregulatory activities within MDSCs include increased production of arginase I [11], inducible NO synthase (iNOS) [12], reactive oxygen species [13, 14], and anti-inflammatory cytokines [15].

The most widely used sources of MDSCs for immunotherapy are the spleen and blood of tumor-bearing mice, but substantial safety considerations must be made in these cases [16]. High concentrations of granulocyte–macrophage colony-stimulating factor (GM-CSF) alone or in combination with other molecules represent the most common ex-vivo protocol to differentiate MDSCs from BM or from peripheral blood mononuclear cells [17]. In this study, we analyzed and characterized the functions of MDSCs under the influence of mouse mesangial cells (MMCs) in a hyperglycemic environment. Additionally, we investigated how cytokine-induced MDSCs (cMDSCs) may ameliorate renal glomerulosclerosis in diabetic mice.

## Methods

### Animals, cell line, and streptozotocin-induced diabetic mice

Male C57BL/6 (B6) and BALB/c mice were purchased from the National Laboratory Animal Center (Taiwan). The mice (B6) were injected intraperitoneally with a single dose of 180 mg/kg streptozotocin (STZ). Those with blood glucose concentrations exceeding 350 mg/dl for 2 consecutive days were considered diabetic mice. All animal experiments were approved by the Institutional Animal Care and Use Committee of the Chang Gung Memorial Hospital (IACUC permit number: 2012091902) and were performed in accordance with the Animal Protection Law by the Council of Agriculture, Executive Yuan (Taiwan) and the National Research Council’s Guide for the Care and Use of Laboratory Animals (USA). The MMC cell line SV40 MES 13, which was derived from ATCC CRL-1927 and obtained from the Bioresource Collection and Research Center (Taiwan), was used in this study.

### Culture of conventional MDSCs and cytokine-induced MDSCs

Bone marrow cells (2 × 10^6^ cells/well) from the tibias and femurs of B6 mice were cultured in Roswell Park Memorial Institute (RPMI)-1640 medium containing 10% fetal calf serum in the presence of mouse recombinant GM-CSF (10 ng/ml; R&D Systems, Minneapolis, MN, USA) for 7 days. Cells that were double positive for CD11b and Gr-1 were considered MDSCs. Cytokine-induced MDSCs were propagated from BM cells cultured with mouse recombinant GM-CSF (10 ng/ml), IL-6 (10 ng/ml; R&D Systems), and IL-1β (10 ng/ml; R&D Systems) for 7 days. The purity of cytokine-induced MDSCs (CD11b^+^/Gr-1^+^) was more than 90% (Additional file 1: Figure S1).

### MMCs cocultured with MDSCs and cytokine production assay

To test the impact of MMCs on the development of MDSCs, MMCs were added at the beginning of the MDSC culture at a MMC:BM cell ratio of 1:80. Floating cells were harvested, washed, and resuspended in RPMI-1640 medium in the presence of mouse recombinant GM-CSF (10 ng/ml) for 7 days. Cells that were double positive for CD11b and Gr-1 were considered MDSCs. Expression of chemokines, growth factors, and immunomodulators in conditioned medium from MMCs (4 × 10^5^ cells/well) cultured at normal glucose (5 mM) or high glucose (25 mM) levels for 72 h was measured using a cytokine array kit.

### Adoptive transfer of cMDSCs into STZ-induced diabetic mice

Cytokine-induced MDSCs (1 × 10^7^ cells) were adoptively transferred into STZ-treated diabetic mice through the pudendal vein once a week for 3 weeks until mice in each group were sacrificed on day 27. Blood sugar levels and body weight were measured twice a week until the mice were sacrificed. Blood sugar was measured from the tail tip in the untreated, STZ-treated, and cMDSC-treated STZ mice using a OneTouch UltraEasy (Johnson-Johnson, New Brunswick, NJ, USA) monitor.

### Flow cytometric analysis

Monoclonal antibodies (mAbs) against CD4, CD11b, CD25, CD40, CD80, CD86, F4/80, Gr-1, and I-Ab (MHC class II) were purchased from BD PharMingen (San Diego, CA, USA), and mAbs against B7-H1 and Foxp3 were purchased from eBioscience (San Diego, CA, USA). Intracellular staining protocols for regulatory T cells were followed for Foxp3 staining. For carboxyfluorescein succinimidyl ester (CFSE) labeling, splenic T cells (10^7^/ml) from BALB/c mice were incubated with 0.5 μM of CFSE (Invitrogen, San Diego, CA, USA) for 10 min at room temperature. Flow analyses were performed with a BD FACSCanto II flow cytometer (BD Bioscience, Franklin Lakes, NJ, USA).

### Cytokine antibody array assay

A cytokine antibody array assay was performed with a mouse cytokine array kit (R&D Systems) according to the manufacturer’s protocol. Briefly, culture supernatants from MMCs were collected and centrifuged. After centrifugation, the assay membranes, which had been precoated with capture antibodies, were incubated with the supernatants. The membranes were then washed with wash buffer and a detection antibody was added using streptavidin–horseradish peroxidase (HRP) and Chemi Reagent Mix. The immunoblot images were captured and visualized using the BioSpectrum Imaging System (Ultra-Violet Products, Ltd, Cambridge, UK) and the intensity of each spot in the captured images was analyzed using ImageQuant 5.0 software (Molecular Dynamics).

### Quantitative reverse transcription PCR

Total RNA was extracted with an RNeasy Mini Kit (Qiagen, Valencia, CA, USA). RNA samples were first converted into cDNA using a RevertAid First Strand cDNA Synthesis Kit (Thermo Fisher, Waltham, MA, USA). For quantitative PCR, the primers were: arginase 1, forward CACGG CAGTG GCTTT AACCT and reverse TGGCG CATTC ACAGT CACTT; iNOS, forward TGGCC ACCTT GTTCAG CTACG and reverse GCCAA GGCCA AACAC AGCAT AC; fibronectin, forward GCTCA GCAAA TCGTG CAGC and reverse CTAGG TAGGT CCGTT CCCAC T; collagen type IV, forward AAAGG GAGAA AGAGG CTTGC and reverse CCTTT GTACC GTTGC ATCCT; and alpha-smooth muscle actin (α-SMA), forward ATGGC TCTGG GCTCT GTAAG and reverse TCTGG GACGT CCCAC GATGGA. mRNAs were measured using a CFX96 Touch Real Time PCR system (Bio-Rad Laboratories, Inc., Hercules, CA, USA) in duplicate and were normalized to 18S mRNA.

### Immunofluorescence staining and confocal microscopy

Tissue samples were embedded in optimal cutting temperature compound (OCT) and snap frozen in liquid nitrogen. Tissue sections (4 μm) were fixed in ethanol/acetic acid fixative solution for 2–10 min, and then were stained with anti-fibronectin (Abcam), CD11b (BD Biosciences), Gr-1 (BD Biosciences), DAPI (Invitrogen), and Alexa Fluor 488 Phalloidin (Invitrogen) overnight at 4 °C in a humidified chamber. After three washes, slides were stained with DAPI and mounted with ProLong Gold mounting medium (Invitrogen). Confocal imaging was performed using a Leica SP5 II confocal microscope.

### Immunohistochemistry

Extracellular matrix expression of the renal cortex in cryostat sections was identified by fluorescent staining using a specific anti-fibronectin (Abcam), anti-collagen type IV (Abcam), anti-alpha-smooth muscle actin (Abcam), anti-Gr1 (BD Biosciences) antibody, following permeabilization with 0.05% saponin buffer using a Vectastain Elite ABC kit (Vector Lab, Inc., Burlingame, CA, USA) as immunoperoxidase. The slides were developed using AEC Chromogen/Substrate and counterstained with hematoxylin. Isotype and species-matched irrelevant antibodies served as controls.

### Western blot analysis

Protein extracts were separated by SDS-PAGE and transferred onto PVDF membranes before being probed with antibodies against fibronectin (15613-1-AP; Proteintech, Chicago, IL, USA) or GAPDH (sc-32,233; Santa Cruz Biotechnology, Santa Cruz, CA, USA). Proteins of interest were detected with an HRP-conjugated goat anti-mouse IgG antibody (sa00001-1; Proteintech) and visualized with a Pierce ECL Western blotting substrate (Thermo Scientific, Rockford, IL, USA) according to the provided protocol.

### Blood and urine analysis

Blood glucose was determined using a OneTouch UltraEasy (Johnson-Johnson) monitor. Blood and urine were collected for examination of serum creatinine and protein when the mice were sacrificed, and serum creatinine and urinary protein were determined using a Labospect 008 (Hitachi, Tokyo, Japan).

### Statistical analyses

Statistical analyses were performed using Student’s *t* test for independent samples, with significance determined at *P* < .05. All data, means, and standard deviations (SDs) were calculated and graphed in Microsoft Excel (Microsoft, Redmond, WA, USA).

## Results

### High extracellular matrix expression of renal cortex and MDSC redistribution in STZ-treated diabetic mice

**Fig. 1:**
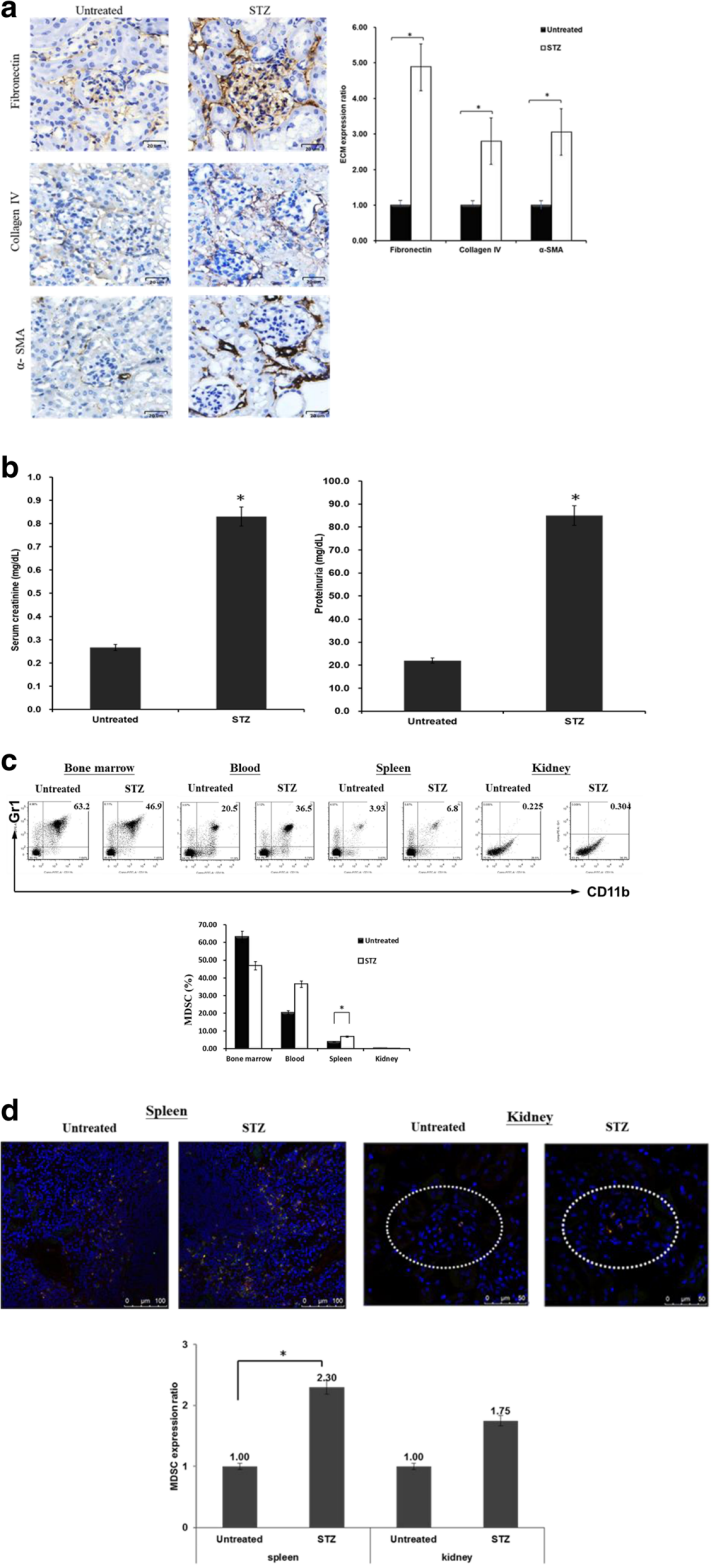
Renal ECM expression and MDSC distribution in STZ-treated diabetic mice. **a** Diabetes induced in mice using one dose of streptozotocin (STZ, 180 mg/kg) intraperitoneally, and blood glucose levels maintained over 350 mg/dl. Four weeks later, mice were sacrificed and ECM expression in kidney examined. Kidney cryostat sections histochemically stained with anti-fibronectin mAb, anti-collagen type IV mAb, or anti-alpha smooth muscle actin mAb (left panel, brown, 400× magnification). Bar graph shows quantification of differences in ECM expression of kidney between STZ-treated diabetic mice and untreated mice (right panel, *P < .05). **b** Blood and urine collected for serum creatinine and protein analyses when mice sacrificed. Level of differences in serum creatinine and proteinuria between STZ-treated diabetic mice and untreated mice (*P < .05). **c** MDSC ratios (CD11b^+^/Gr-1^+^) in BM, blood, spleen, and kidneys of STZ-treated diabetic mice and untreated mice compared. Isolated cells were two-color stained with specific mAbs against CD11b and Gr-1 for flow analyses. Double-positive CD11b and Gr-1 cells represent MDSCs (*P < .05). **d** Cryostat sections of spleen and kidney from STZ-treated diabetic mice and untreated mice double-stained with anti-CD11b (green) and anti-Gr-1 (red) mAbs and evaluated under fluorescent microscope (400× magnification; upper panel). Double-positive cells counted. In total, 10 high-power fields randomly selected in each section. Data expressed as mean CD11b^+^/Gr-1^+^ cells ± 1 SD (lower panel, *P < .05). Data representative of three separate experiments. α-SMA alpha-smooth muscle actin, ECM extracellular matrix, MDSC myeloid-derived suppressor cell

Diabetes is a proinflammatory state. ECM accumulation in the renal parenchyma plays a pivotal role in the pathogenesis of diabetic nephropathy. In our study, ECM—such as fibronectin, collagen type IV, and alpha-smooth muscle actin (α-SMA)—in the renal cortex was more highly expressed in the STZ-treated mice than in the untreated mice (Fig. 1a, *P* < .05). Fibronectin was primarily accumulated in the glomerulus, whereas collagen type IV and α-SMA were largely gathered in the renal tubules. Serum creatinine (0.83 ± 0.18 vs 0.27 ± 0.05 mg/dl) and urinary protein (85.00 ± 10.00 vs 22.00 ± 6.56 mg/dl) levels were significantly higher in the STZ-treated mice than in the untreated mice (Fig. 1b, *P* < .05).

MDSCs are heterogeneous immature myeloid cells that rapidly expand to regulate host immunity during inflammation, infection, and cancer. The distribution of MDSCs within a hyperglycemic environment was investigated through in-vivo assays. As shown in Fig. 1c, the number of MDSCs in STZ-treated mice was lower in the BM (46.9% vs 63.2%, *P* = .16) than in the untreated mice, whereas the ratios of MDSCs increased in the peripheral blood (36.5% vs 20.5%, *P* = .07), spleen (6.8% vs 3.93%, *P* = .03), and kidneys (0.304% vs 0.225%, *P* = .14). As an inflammatory state, diabetes may trigger the redistribution of MDSCs from the BM to peripheral organs, including the peripheral blood, spleen, and kidneys. Similar results of MDSC expansion were also noted in the spleen parenchyma (Fig. 1d, upper panel), and a slight increase was observed in the renal glomerulus (Fig. 1d, upper panel, dotted circle) in the STZ-treated mice through immunofluorescence staining. The numbers of MDSCs within the spleen parenchyma and renal glomerulus in the STZ-treated mice were 2.3 and 1.75 times that of untreated mice (*P* < .05 and *P* = .183, respectively; Fig. 1d, lower panel). Together, these results demonstrated that higher ECM expression occurs in the renal cortex, and that MDSCs are redistributed from the BM to the peripheral organs in STZ-treated diabetic mice.

### Hyperglycemic MMCs produce more fibronectin and proinflammatory cytokines

**Fig. 2:**
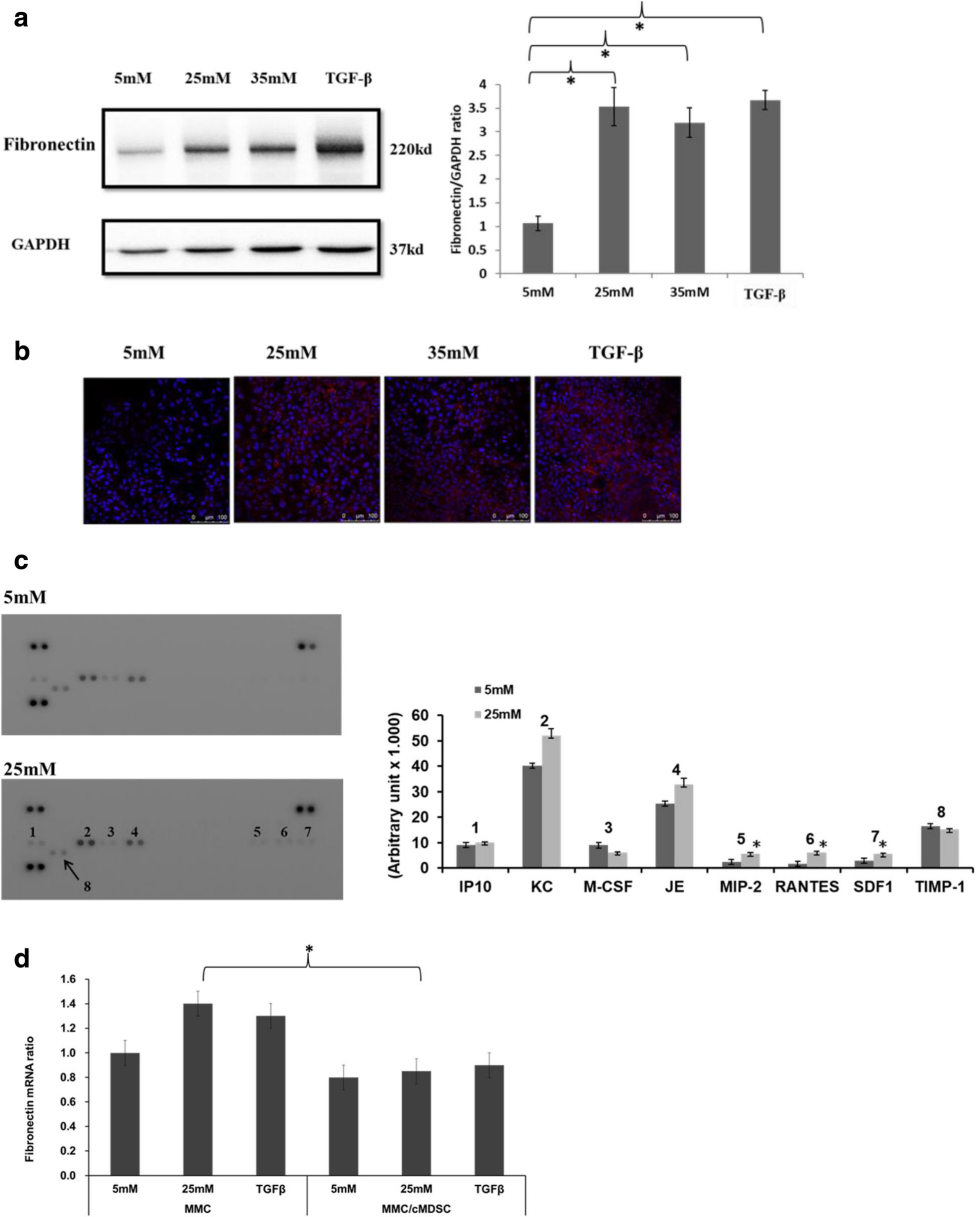
MMCs produced more fibronectin and inflammatory cytokines in hyperglycemic environment. **a** Fibronectin protein expression, assessed through western blotting, in MMCs exposed to 5 mM, 25 mM, and 35 mM of glucose as well as TGF-β (2 ng/ml) with 5 mM of glucose for 24 h. Quantitative ratios of fibronectin expression compared with that of GAPDH (*P < .05). **b** Fibronectin expression pattern of MMCs compared through immunofluorescence staining (red for fibronectin, blue for nuclear DAPI stain; 400× magnification). **c** Expression of chemokines, growth factors, and immunomodulators in conditioned medium from MMCs cultured in normal (5 mM) or high glucose (25 mM) levels for 72 h using a cytokine array kit. Bar graph constructed for each cytokine using median value in arbitrary units of three independent assays (*P < .05). **d** Expression of fibronectin mRNA ratios from MMCs cocultured with or without cMDSCs at ratio of 1:4 in different glucose concentrations or stimulated with TGF-β for 24 h determined using qPCR (*P < .05). MMCs cocultured with cMDSCs assayed using Transwell method (top layer, cMDSC; bottom layer, MMC). GAPDH glyceraldehyde 3-phosphate dehydrogenase, IP10 interferon gamma-induced protein 10, JE CCL2, KC CXCL1, M-CSF macrophage colony-stimulating factor, MDSC myeloid-derived suppressor cell, MIP-2 macrophage inflammatory protein 2, MMC mouse mesangial cell, RANTES regulated on activation, normal T cells expressed and secreted, SDF-1 stromal-cell-derived factor-1, TGF-β transforming growth factor beta, TIMP-1 tissue inhibitors of metalloproteinases

Mesangial cells are specialized cells that accumulate in the glomerular mesangium and, together with mesangial matrix, form the vascular pole of the glomerulus. These cells play a crucial role in the process of glomerulosclerosis in diabetic nephropathy. As shown in Fig. 2a, fibronectin protein expression was found to be significantly higher in MMCs under hyperglycemic conditions and in MMCs stimulated with transforming growth factor beta (TGF-β) cytokine compared with MMCs under normal glucose concentrations (5 mM, *P* < .05). Through immunofluorescence staining, a similar result was observed in MMCs that expressed higher levels of fibronectin when cultured under hyperglycemic conditions or when stimulated with TGF-β cytokine (Fig. 2b).

To identify differences in cytokines released from MMCs between normal glucose (5 mM) and high glucose (25 mM) levels, a cytokine array assay was conducted. Multiple cytokines were detected in MMC medium incubated with 5 mM or 25 mM of glucose. Cytokines IP10, KC, JE, MIP-2, RANTES, and SDF-1 tended to exhibit higher expression levels in the 25-mM group. Among those identified, MIP-2, RANTES, and SDF-1 levels were significantly higher in the 25-mM group (Fig. 2c, *P* < .05) than in normal glucose conditions.

According to the aforementioned results, MMCs produced more fibronectin and proinflammatory cytokines under hyperglycemic conditions than did MMCs under standard conditions. MDSCs have been reported to participate in immune suppression and autoimmune disorders. Specifically, the adoptive transfer of MDSCs prevented autoimmune arthritis in mouse models [18]. To examine the influence of MDSCs on MMCs in a hyperglycemic environment, cytokine-induced MDSCs were propagated and cocultured with MMCs using a Transwell assay. As illustrated in Fig. 2d, MMCs decreased the production of fibronectin mRNA in the presence of cMDSCs, especially when the mice were in a hyperglycemic state (25 mM, *P* = .04). These results suggest that MMCs produce elevated levels of fibronectin and proinflammatory cytokines under hyperglycemic conditions and that cMDSCs attenuate fibronectin production in vitro.

### cMDSCs ameliorate renal fibronectin expression in STZ-treated diabetic mice

**Fig. 3:**
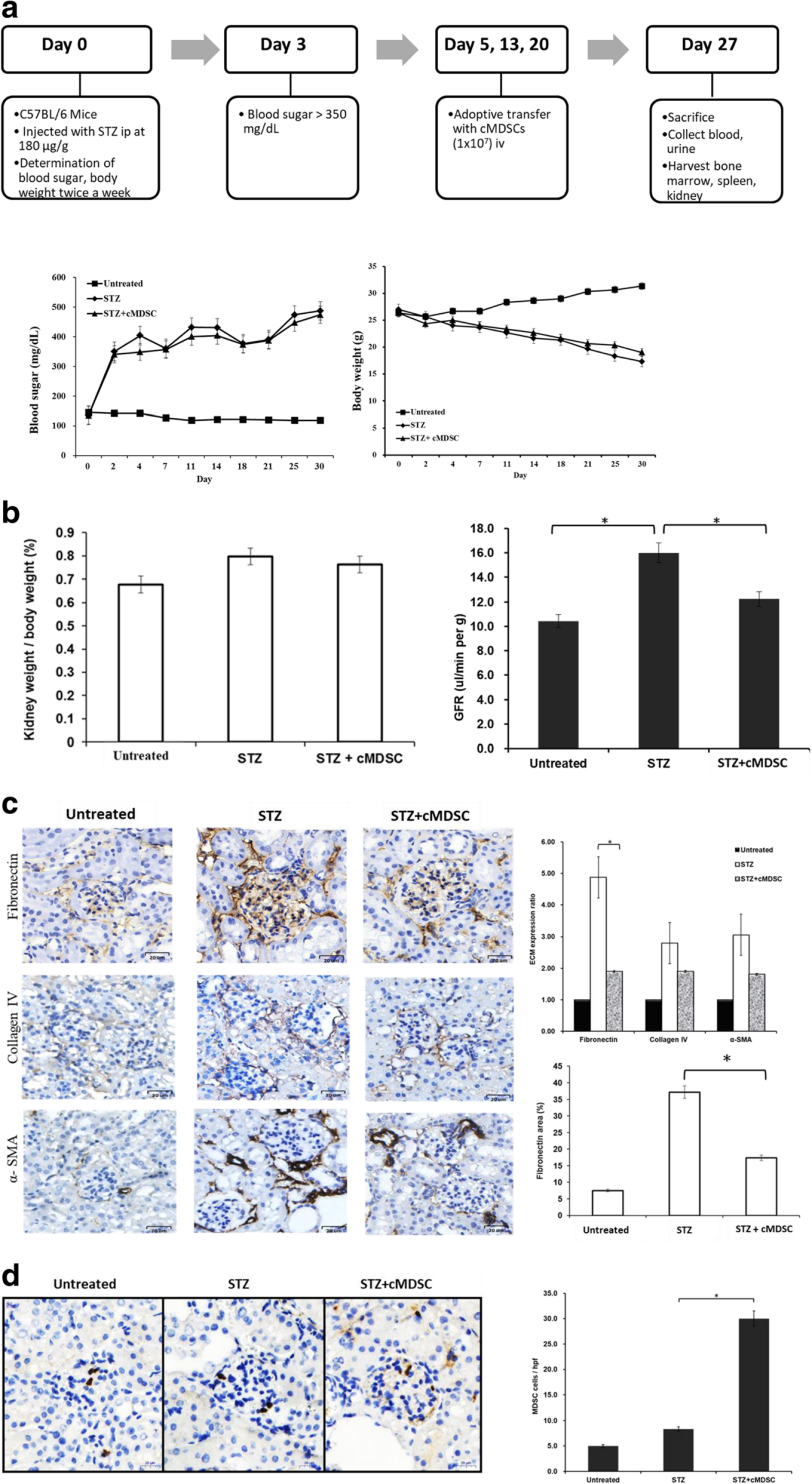
Adoptive transfer of cMDSCs into STZ-treated diabetic mice. **a** Cytokine-induced MDSCs (1 × 10^7^ cells) adoptively transferred into STZ mice through pudendal vein once a week (on days 5, 13, and 20). Mice in each group then sacrificed on day 27 (upper panel). Blood sugar levels and body weight (lower panel) measured twice a week until mice sacrificed in subgroups of untreated mice (untreated, *n* = 5), STZ-treated diabetic mice (STZ, *n* = 5), and cMDSC-treated STZ mice (STZ + cMDSC, *n* = 5). **b** Kidneys harvested and weights measured on day 27. Ratios of kidney weight to body weight of the three groups compared (left panel). GFRs of the three groups examined and compared (right panel) (*P < .05). **c** Kidney cryostat sections histochemically stained with anti-fibronectin mAb, anti-collagen type IV mAb, or anti-alpha-smooth muscle actin mAb (left panel, brown; 400× magnification) and examined using a microscope. Bar graph presents quantification of ECM expression of renal cortex of untreated mice, STZ mice, and cMDSC-treated STZ mice (right upper panel) (*P < .05). Areas of fibronectin expression within the glomeruli of the three groups quantified (right lower panel) (*P < .05). **d** Presence of MDSCs in kidneys analyzed through histochemical staining with anti-Gr-1 mAb (left panel, brown; 400× magnification) and examined using a microscope. Bar graph illustrates positive MDSCs counted in a total of 10 high-power fields randomly selected in each section (right panel) (*P < .05). α-SMA alpha-smooth muscle actin, cMDSC cytokine-induced myeloid-derived suppressor cell, ECM extracellular matrix, GFR glomerular filtration rate, iv intravenous, STZ streptozotocin

To examine whether the anti-inflammatory effect of MDSCs influenced the production of renal fibronectin in diabetic mice, cMDSCs were adoptively transferred into STZ-treated mice (Fig. 3a, upper panel). As presented in Fig. 3a, blood sugar levels were higher in the cMDSC-treated and untreated STZ mice than in the untreated control mice (Fig. 3a, lower left panel). Similarly, body weight was reduced in both STZ groups in comparison with the untreated group (Fig. 3a, lower right panel). The adoptive transfer of cMDSCs did not change the blood sugar and body weight of diabetic mice. However, the kidney weight in proportion to body weight was higher in STZ mice compared with untreated mice, but treatment with cMDSCs reduced the magnitude of these differences (Fig. 3b, left panel, *P* > .05). The glomerular filtration rate (GFR) is the optimal test for measuring kidney function and determining the kidney disease stage. The level of GFR was elevated in STZ mice compared with untreated mice (16.00 ± 2.00 vs 10.43 ± 1.40 μl/min/g, *P* = .004), whereas this level was reduced after the administration of cMDSCs into STZ mice (16.00 ± 2.00 vs 12.23 ± 1.12 μl/min/g, *P* = .020) (Fig. 3b, right panel).

Glomerulosclerosis in patients with diabetic nephropathy is caused by the accumulation of ECM proteins in the mesangial interstitial space. Fibronectin is a crucial component of ECM proteins. In our study, fibronectin production in the renal glomerulus was significantly increased in STZ mice, but the administration of cMDSCs ameliorated this abnormal fibronectin and reduced the resulting glomerular area accumulation in STZ mice (Fig. 3c, *P* < .05). In the other two groups, ECM, collagen type IV, and α-SMA decreased the production of fibronectin after treatment with cMDSCs, but no statistical difference was observed (*P* > .05). The number of MDSCs within the glomerulus was considerably enhanced in the STZ mice receiving cMDSC treatment (Fig. 3d). Taken together, these data indicate that cMDSCs exhibit anti-inflammatory properties that substantially improve fibronectin accumulation in the renal glomerulus and normalize the GFR in STZ-treated diabetic mice.

### MMCs reduce MDSC production and function under hyperglycemic conditions in vitro

The influence of components from the blood, especially circulating immune cells, on mesangial cells may contribute to the pathogenesis of glomerulosclerosis. To examine the interaction between mesangial and immune cells we set up an in-vitro culture system to generate MDSCs (CD11b^+^/Gr1^+^) through coculturing MMCs with BM-derived monocytes under normal (5 mM) or high (25 mM) glucose levels.

**Fig. 4:**
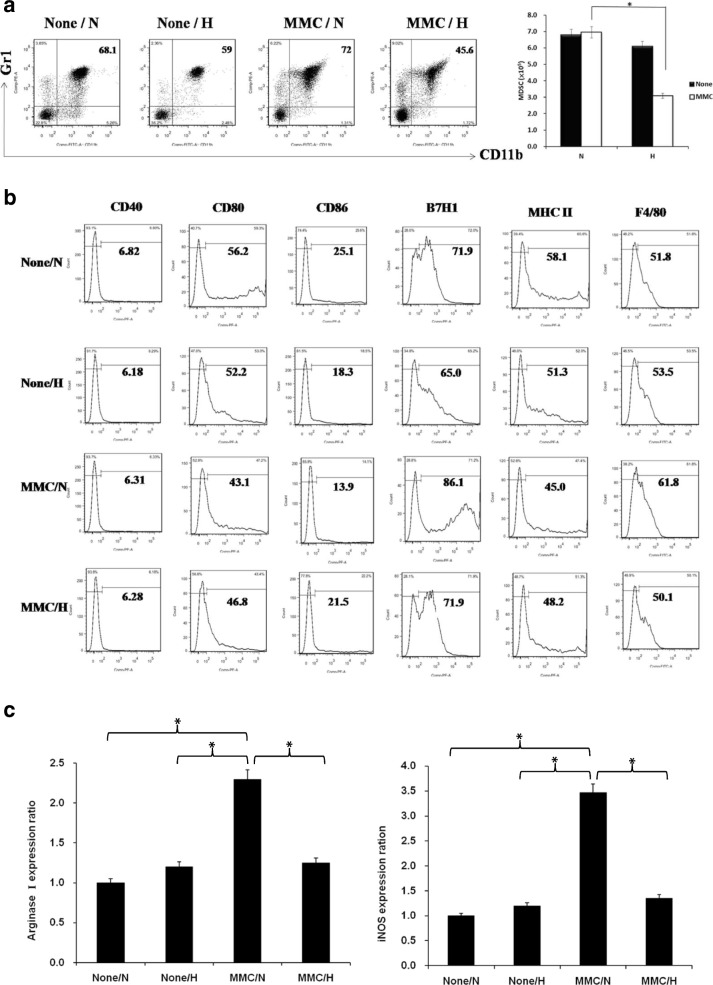
Effects of MMCs on development of MDSCs under hyperglycemic conditions. BM cells from femur and tibia of B6 mice mixed with or without MMCs in presence of mouse recombinant GM-CSF (10 ng/ml) at ratio of 80:1 under normal (N) (5 mM, 90 mg/dl) or high (H) (25 mM, 450 mg/dl) glucose conditions. Cells isolated for examination 7 days later. **a** Isolated cells two-color stained with specific mAbs against CD11b and Gr-1 for flow analyses. Four groups divided based on presence or absence of MMCs and glucose levels. Production of MDSCs from different cultured systems measured. Double-positive CD11b and Gr-1 cells represent MDSCs. Bar graph displays yield of MDSCs for each group. Data expressed as mean CD11b^+^/Gr-1^+^ cells ± 1 SD (*P < .05). **b** Cell surface molecules stained with specific mAbs against CD40, CD80, CD86, B7H1, MHC class II, and F4/80. Numbers represent percentage of positive cells. **c** Expression of arginase 1 and iNOS mRNA from MDSCs determined using qPCR (*P < .05). Data representative of three separate experiments. iNOS inducible nitric oxide synthase, MDSC myeloid-derived suppressor cell, MMC mouse mesangial cell

Compared with normal glucose levels, MDSC production was slightly decreased in a high-glucose environment and slightly increased when cocultured with MMCs at a normal glucose level (Fig. 4a, left panel). By contrast, the production of MDSCs was substantially reduced when cocultured with MMCs under hyperglycemic conditions (Fig. 4a, right panel). This result demonstrated that MMCs probably attenuate MDSC development under hyperglycemic conditions. The effect of D-mannitol didn't influence MDSCs production (Additional file 2: Figure S2). MDSCs expressed less costimulatory CD80, CD86, and MHC class II and more inhibitory B7H1 under the influence of MMCs in the normal glucose environment (Fig. 4b), indicating that MMC-directed MDSCs exhibit a greater capacity to suppress the immune response than do MDSCs alone. By contrast, MMC-directed MDSCs weaken the immunomodulatory ability of these cells in a hyperglycemic environment.

Arginase I and inducible NO synthase (iNOS) are known to participate in the immunoregulatory activities of MDSCs. In our study, MMC-directed MDSCs in normal glucose conditions expressed the highest levels of arginase 1and iNOS among the four cultured groups (Fig. 4c, *P* < .05). In high-glucose conditions, MDSCs mediated by MMCs exhibited significantly reduced immunosuppressive properties compared with those of MMC-directed MDSCs in normal glucose conditions. Taken together, these results suggest that MMCs diminished the production of MDSCs and their immunosuppressive function in a hyperglycemic environment.

### Hyperglycemic MMC-directed MDSCs modulate adaptive immunity and induce an inflammatory state

**Fig. 5:**
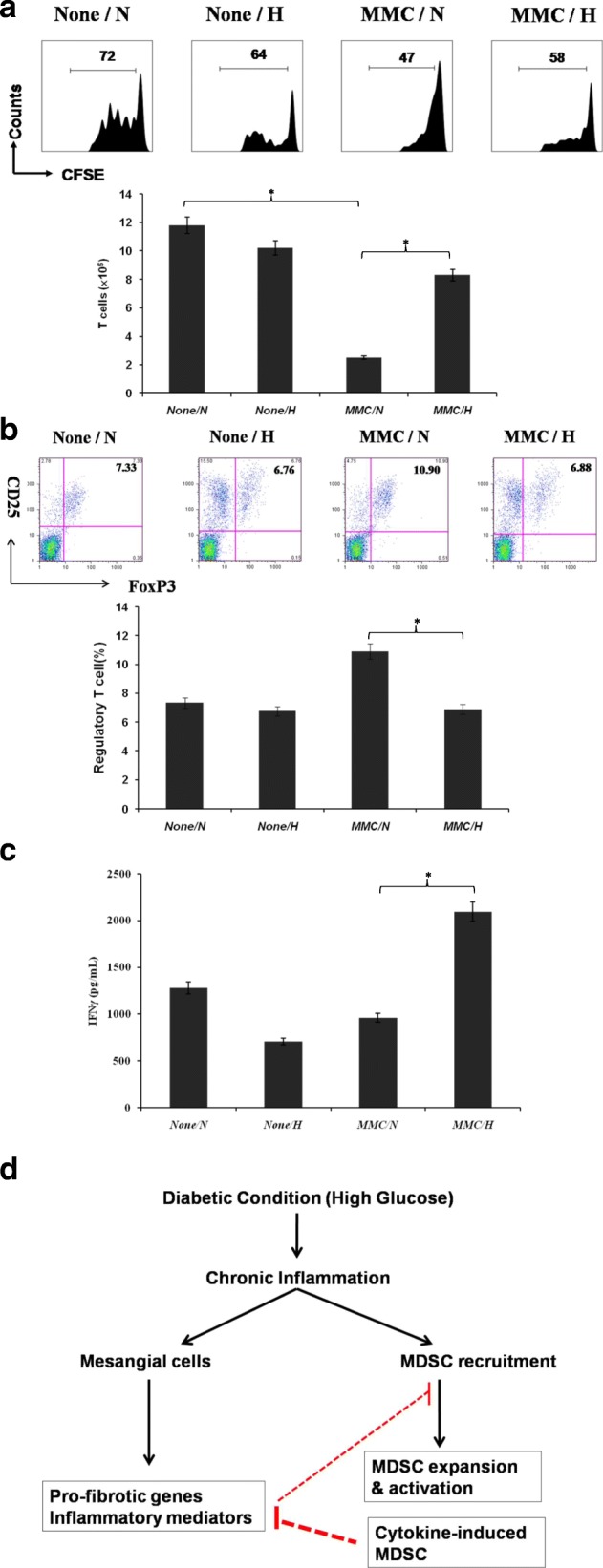
Effects of MDSCs on T-cell development and differentiation. **a** Antigen-presenting activity of MDSCs. MDSCs cultured with CFSE-labeled BALB/c spleen T cells at a ratio of 1:20 for 3 days. Proliferative response determined by CFSE dilution (upper panel). Bar graph illustrates T-cell production from four types of MDSCs (lower panel) (*P < .05). **b** Expression of regulatory T cells (CD4^+^/CD25^+^/FoxP3^+^) assayed through intracellular staining with specific mAbs and analyzed using flow cytometry. Numbers represent percentage of double-positive cells in CD4^+^ T-cell subset (upper panel). Bar graph shows ratio of regulatory T cells differentiated from four types of MDSCs (lower panel) (*P < .05). **c** IFNγ production of MDSC-stimulated T cells. Cytokine levels of IFNγ analyzed from supernatant of activated T cells through ELISA (*P < .05). **d** Model for interaction between renal stromal cells and MDSCs during chronic inflammation of diabetic conditions. CFSE carboxyfluorescein diacetate succinimidyl ester, H high glucose, IFNγ interferon gamma, MDSC myeloid-derived suppressor cell, MMC mouse mesangial cell, N normal glucose

Members of the MDSC-expressed MHC class II (Fig. 4b) proteins are normally found only on antigen-presenting cells that initiate adaptive immune responses. To verify the functions of MDSCs and the influence of adaptive immunity, especially in T cells, an alloreactive T-cell proliferation and differentiation assay as well as a cytokine production assay were performed. MMC-conditioned MDSCs induced more allogeneic T-cell activation in a hyperglycemic state than at normal glucose levels (Fig. 5a, upper panel). The total T-cell counts after the introduction of MMC-conditioned MDSCs were considerably higher in the mice subjected to the hyperglycemic environment than those in the normal glucose conditions (Fig. 5a, lower panel, *P* < .05).

Regulatory T cells (CD4^+^/CD25^+^/FoxP3^+^) are a subpopulation of T cells that modulate the immune system, maintain tolerance to self-antigens, and prevent autoimmune disease. The induction of regulatory T cells is a possible mechanism by which MDSCs suppress the host’s proinflammatory immunity. In our study, MMC-directed MDSCs elicited greater regulatory T-cell differentiation in normal glucose levels than in hyperglycemic conditions (Fig. 5b, *P* < .05). More interferon gamma (IFNγ), a proinflammatory cytokine, was secreted from T cells stimulated by MMC-conditioned MDSCs under the hyperglycemic conditions than MDSCs in the normal glucose environment (Fig. 5c, *P* < .05). These data reveal that MDSCs conditioned by MMCs in a hyperglycemic state had a reduced inhibitory immune response, and an inflammatory environment was created. A model for the interaction between renal stromal cells and MDSCs during chronic inflammation under diabetic conditions is proposed in Fig. 5d.

## Discussion

Diabetic nephropathy is currently the most common cause of end-stage renal disease worldwide [2]. Glomerulosclerosis is a key contributor to diabetic nephropathy. Glomerulosclerosis is caused by accumulation of ECM proteins in the mesangial interstitial space, resulting in fibrosis [3]. Mesangial cells are unique stromal cells that account for approximately 30–40% of the total cells in the renal glomerulus [19] and form the vascular pole of the renal corpuscle with the mesangial matrix [20]. The influence of the circulating immune cells on mesangial cells may contribute to the pathogenesis of glomerulosclerosis in diabetic nephropathy.

Understanding the population dynamics of MDSCs during diabetes progression is a crucial step in elucidating the possible role of MDSCs in diabetic nephropathy. In this study, STZ-treated mice had an increased proportion of MDSCs in the peripheral blood, spleen, and kidneys, whereas a decrease was noted in the relative number of MDSCs in the BM. This was likely caused by the inflammatory diabetic state, which could have triggered immature myeloid cells to be redistributed from the BM into peripheral organs. The ratio of MDSCs (63.2%) within BM cells in healthy mice here was higher than that was approximately 30% of BM cells in other study [21]. The possible reasons are the heterogeneity of MDSCs, including immature granulocytes, macrophages, and dendritic cells, and the sensitivity of monoclonal antibodies to CD11b and Gr-1. The function of splenic MDSCs isolated from STZ-treated mice was less inhibitory to T-cell proliferation than those isolated from untreated mice (Additional file 3: Figure S3). Whitfield-Larry et al. [22] reported an increase in MDSCs in the peripheral blood and spleen, and a reduction of such cells in the pancreatic islets of nonobese diabetic (NOD) mice. The functions of native MDSCs in NOD mice are not as maximally suppressive of T-cell proliferation compared with those in in-vitro conditions induced by IL-1β and GM-CSF cytokines. MDSCs are redistributed from the BM into peripheral organs and have a reduced suppressive activity in diabetic mice.

MMCs produced more fibronectin and proinflammatory cytokines, such as MIP-2, RANTES, and SDF-1, under hyperglycemic conditions. Fibronectin has a crucial role in the organization of ECM components and the pathogenesis of glomerulosclerosis in diabetic nephropathy [23]. The TGF-β/mothers against decapentaplegic homolog (SMAD) axis is arguably the most critical signaling pathway involved in fibronectin production in mesangial cells [24]. MIP-2 is a chemokine and chemoattractant for polymorphonuclear leukocytes that is involved in inflammatory processes [25]. The chemokine of RANTES (CCL5) is chemotactic for T cells, eosinophils, and basophils, and plays an active role in recruiting leukocytes into inflammatory sites, as well as promoting cancer progression [26]. SDF-1 is also chemotactic for mesenchymal stem cell migration in response to inflammatory stimuli [27]. Collectively, both the major components of ECM and proinflammatory cytokines secreted from renal stromal cells under hyperglycemic conditions enhance fibrotic changes in the glomerulus.

MDSCs are heterogeneous immature myeloid cells that rapidly expand to regulate host immunity during inflammation, infection, and cancer. Immunoregulatory activities within MDSCs include increased production of arginase I, inducible NO synthase, reactive oxygen species, and anti-inflammatory cytokines [28]. Investigating the interaction between renal stromal cells and circulating immune cells, such as MDSCs, is a critical step in elucidating the mechanisms of glomerulosclerosis in diabetic nephropathy. In this study, we assembled an in-vitro culture system to generate MDSCs (CD11b^+^/Gr1^+^) by coculturing MMCs with BM-derived monocytes. The production of MDSCs was significantly reduced when BM cells were cocultured with MMCs under hyperglycemic conditions (25 mM) compared with a normal glucose environment (5 mM). The results demonstrated that mesangial cells attenuated MDSC development in a hyperglycemic environment.

MDSCs expressed more costimulatory CD86 and less inhibitory B7H1 as well as lower arginase I and iNOS levels under the influence of MMCs in a high-glucose environment compared with those propagated with MMCs in normal glucose conditions. MDSCs are antigen-presenting cells that initiate adaptive immunity. In the present study, hyperglycemic MMC-directed MDSCs induced more allogeneic T cells, less regulatory T cells, and higher IFNγ levels than did normoglycemic MMC-induced MDSCs. Taken together, MDSCs conditioned by MMCs in a hyperglycemic state exhibited diminished inhibitory immune activities and created an inflammatory environment in the in-vitro cocultured assay.

Immunotherapy provides an alternative treatment protocol for patients with cancer or inflammation [29, 30] and those requiring organ transplantation [31, 32]. MDSCs have been extensively used for immunotherapy based on their immunosuppressive and anti-inflammatory activities. The most widely used sources of MDSCs originate from tumor-bearing mice [16] and ex-vivo procedures with GM-CSF-based cytokines [17, 33]. Differentiating MDSCs using ex-vivo procedures is a feasible and safe method compared with MDSCs propagated from tumor-bearing mice. In this study, cytokine-induced MDSCs were obtained from BM cells cultured with GM-CSF, IL-1β, and IL-6 cytokines. The purity of the cytokine-induced MDSCs was more than 90% in the presence of GM-CSF, IL-1β, and IL-6 cytokines (Additional file 1: Figure S1). The immunoregulatory functions of MDSCs induced by a combination of GM-CSF, IL-1β, and IL-6 have more intense activities than those induced by GM-CSF and IL-1β [22] or GM-CSF and IL-6 [33] (Additional file 4: Figure S4). The increased ratio of kidney to body weight and GFR in STZ-treated diabetic mice indicated that hyperfiltration in early onset diabetes causes hypertrophy. The kidney to body weight ratio and the GFR were normalized after treatment with cytokine-induced MDSCs. In addition, the adoptive transfer of cytokine-induced MDSCs into STZ-treated mice reduced fibronectin accumulation in the renal glomerulus, ameliorating renal glomerulosclerosis. These results demonstrate that the anti-inflammatory activities of cytokine-induced MDSCs offer an alternative immunotherapy protocol for patients with diabetic nephropathy.

## Conclusion

The application of cytokine-induced MDSCs provides a promising strategy for the treatment of glomerulosclerosis and the prevention of diabetic nephropathy.

## Additional files

Additional file 1: **Figure S1.** Propagation of cytokine-induced MDSCs. Cytokine-induced MDSCs were propagated from BM cells cultured with mouse recombinant GM-CSF alone, GM-CSF + IL-6, GM-CSF + IL-1β, or GM-CSF + IL-6 + IL-1β for 7 days. Isolated cells two-color stained with specific mAbs against CD11b and Gr-1 for flow analyses. Four groups divided based on different cytokine-cultured conditions. CD11b^+^ and Gr-1^+^ cells represent MDSCs (PDF 141 kb)

Additional file 2: **Figure S4.** Glucose, not d-mannitol, reduced MDSC production. BM cells from femur and tibia of B6 mice were cultured in presence of mouse recombinant GM-CSF (10 ng/ml) under normal or high glucose conditions with or without 20 mM of d-mannitol. Cells isolated for examination 7 days later. Isolated cells two-color stained with specific mAbs against CD11b and Gr-1 for flow analyses (PDF 111 kb)

Additional file 3: **Figure S2.** MDSCs isolated from STZ-treated mice inhibit less T-cell proliferative responses. CFSE-labeled B6 mice spleen T cells were cultured with splenic MDSCs isolated from STZ-treated mice or untreated mice at ratio of 10:1, 20:1, or 40:1 in presence of 1 μg/ml of CD3/CD28 for 3 days. Proliferation of T cells determined by CFSE dilution (PDF 120 kb)

Additional file 4: **Figure S3.** mRNA expression of arginase 1 and iNOS in cytokine-induced MDSCs. Expression of arginase 1 and iNOS mRNA from MDSCs derived from BM cells propagated for 7 days in presence of GM-CSF alone, GM-CSF + IL-1β, GM-CSF + IL6, and GM-CSF+ IL-1β + IL6 determined through qPCR (*P < .05). Data representative of three separate experiments (PDF 53 kb)

## Abbreviations

B7H1: B7 homolog 1
BM: Bone marrow
CD: Cluster of differentiation
CFSE: Carboxyfluorescein succinimidyl ester
cMDSC: Cytokine-induced myeloid-derived suppressor cell
ECM: Extracellular matrix
GAPDH: Glyceraldehyde 3-phosphate dehydrogenase
GFR: Glomerular filtration rate
GM-CSF: Granulocyte–macrophage colony-stimulating factor
IFNγ: Interferon gamma
IL-1β: Interleukin 1 beta
IL-6: Interleukin 6
iNOS: Inducible NO synthase
IP10: Interferon gamma-induced protein 10
JE: CCL2
KC: CXCL1
MDSC: Myeloid-derived suppressor cell
MHC: Major histocompatibility complex
MIP-2: Macrophage inflammatory protein-2
MMC: Mouse mesangial cell
NOD: Nonobese diabetic
OCT: Optimal cutting temperature
PCR: Polymerase chain reaction
RANTES: Regulated on activation, normal T cells expressed and secreted
SDF-1: Stromal-cell-derived factor-1
STZ: Streptozotocin
TGF-β: Transforming growth factor beta
α-SMA: alpha-smooth muscle actin
