# Supplementary figures and images for: Administration of cytokine-induced myeloid-derived suppressor cells ameliorates renal fibrosis in diabetic mice
Source: Stem Cell Res Ther. 2018 Jul 4;9:183. doi: 10.1186/s13287-018-0915-0 (PMC6032782; doi:10.1186/s13287-018-0915-0)

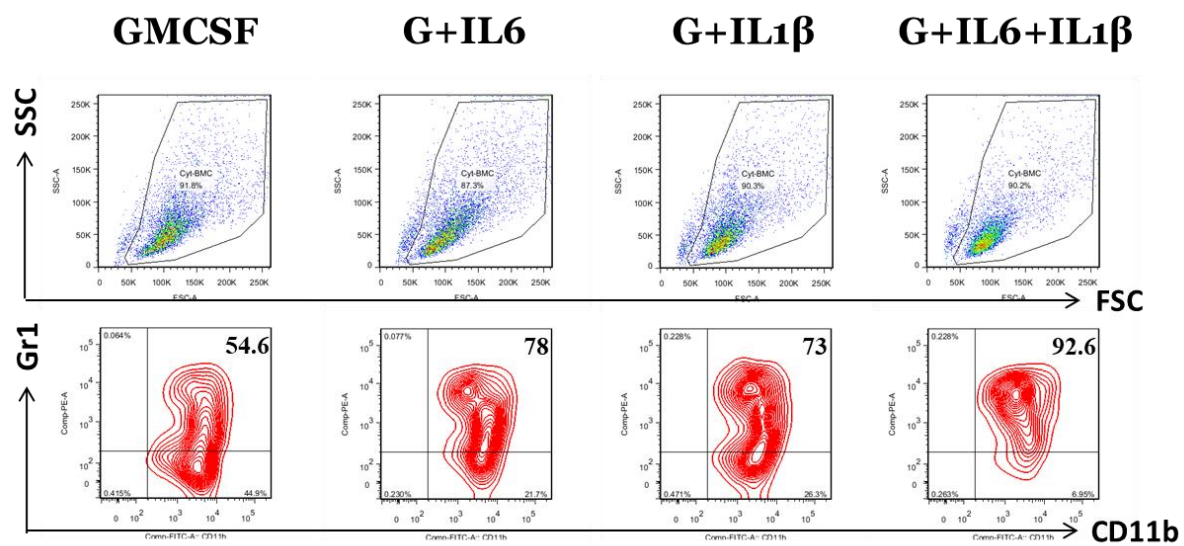

Supplement: Supplementary file 1 — Figure S1. Propagation of cytokine-induced MDSCs. Cytokine-induced MDSCs were propagated from BM cells cultured with mouse recombinant GM-CSF alone, GM-CSF + IL-6, GM-CSF + IL-1β, or GM-CSF + IL-6 + IL-1β for 7 days. Isolated cells two-color stained with specific mAbs against CD11b and Gr-1 for flow analyses. Four groups divided based on different cytokine-cultured conditions. CD11b+ and Gr-1+ cells represent MDSCs (PDF 141 kb) [file 13287_2018_915_MOESM1_ESM.pdf]

Glucose  
D-mannitol

5mM  
0mM

5mM  
20mM

25mM  
0mM

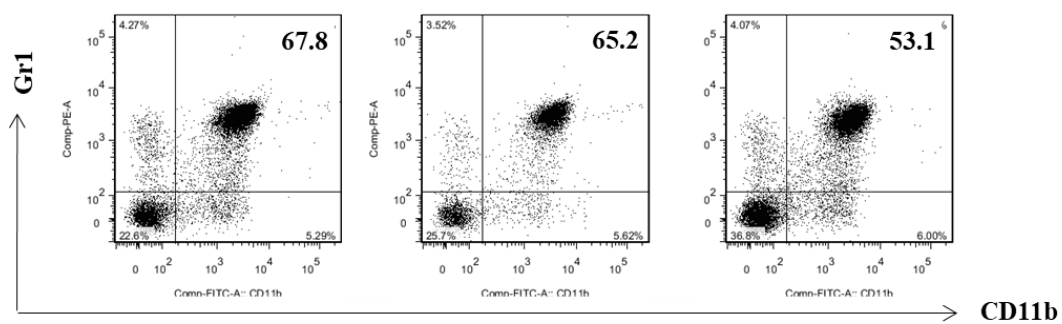

Supplement: Supplementary file 2 — Figure S4. Glucose, not d-mannitol, reduced MDSC production. BM cells from femur and tibia of B6 mice were cultured in presence of mouse recombinant GM-CSF (10 ng/ml) under normal or high glucose conditions with or without 20 mM of d-mannitol. Cells isolated for examination 7 days later. Isolated cells two-color stained with specific mAbs against CD11b and Gr-1 for flow analyses (PDF 111 kb) [file 13287_2018_915_MOESM2_ESM.pdf]

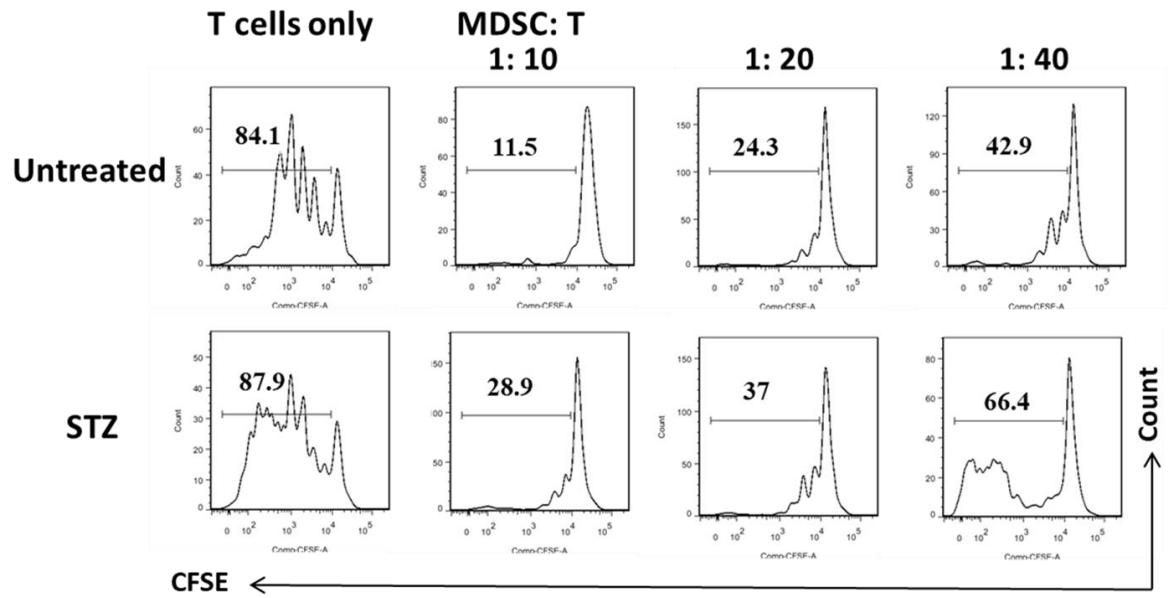

Supplement: Supplementary file 3 — Figure S2. MDSCs isolated from STZ-treated mice inhibit less T-cell proliferative responses. CFSE-labeled B6 mice spleen T cells were cultured with splenic MDSCs isolated from STZ-treated mice or untreated mice at ratio of 10:1, 20:1, or 40:1 in presence of 1 μg/ml of CD3/CD28 for 3 days. Proliferation of T cells determined by CFSE dilution (PDF 120 kb) [file 13287_2018_915_MOESM3_ESM.pdf]

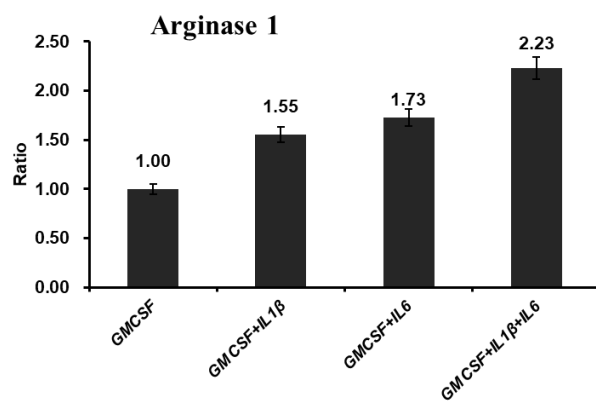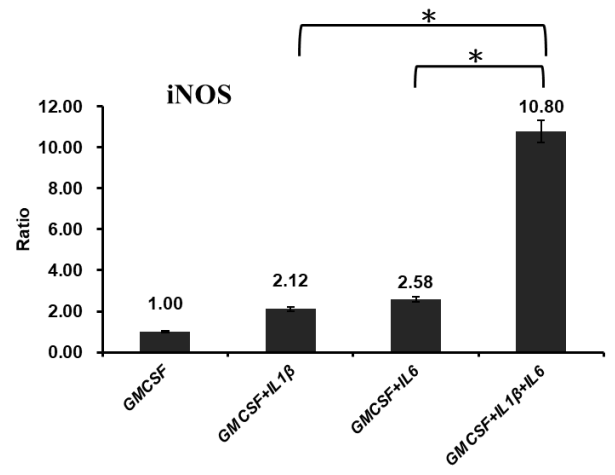

Supplement: Supplementary file 4 — Figure S3. mRNA expression of arginase 1 and iNOS in cytokine-induced MDSCs. Expression of arginase 1 and iNOS mRNA from MDSCs derived from BM cells propagated for 7 days in presence of GM-CSF alone, GM-CSF + IL-1β, GM-CSF + IL6, and GM-CSF+ IL-1β + IL6 determined through qPCR (*P < .05). Data representative of three separate experiments (PDF 53 kb) [file 13287_2018_915_MOESM4_ESM.pdf]
